# Triamcinolone acetonide and anecortave acetate do not stimulate uveal melanoma cell growth

**Published:** 2008-09-24

**Authors:** Mariam el Filali, Irene Homminga, Willem Maat, Pieter A. van der Velden, Martine J. Jager

**Affiliations:** Department of Ophthalmology, Leiden University Medical Centre, Leiden, the Netherlands

## Abstract

**Purpose:**

Radiotherapy-induced radiation retinopathy can develop in over 40% of eyes treated for uveal melanoma. Triamcinolone acetonide (TA) and anecortave acetate (AA) can be used to treat radiation retinopathy. It is not known whether TA or AA has any effect on potentially still viable uveal melanoma cells in the choroid after radiotherapy. We therefore studied the effect of these drugs on the proliferation of uveal melanoma cell lines in vitro. Furthermore, as these drugs are supposed to counteract vascular leakage, we determined their effect on the expression and production of the proangiogenic vascular endothelial growth factor-A (VEGF-A), the antiangiogenic pigment epithelium-derived factor (PEDF), and thrombospondin-1 (TSP-1) in uveal melanoma cells.

**Methods:**

Three uveal melanoma cell lines were treated in vitro with TA or AA. Cell proliferation was measured by counting cells and using the Water-Soluble Tetrazolium Salt-1 (WST-1) assay. VEGF-A and PEDF production was measured by ELISA, and intracellular expression of angiogenic-associated genes including VEGF-A, PEDF, and TSP-1 was determined by real-time quantitative RT–PCR.

**Results:**

We found no effect of TA or AA on tumor cell growth or production of VEGF-A and PEDF in any of the three uveal melanoma cell lines tested. Regarding expression as measured by RT–PCR, TA had an inhibiting effect on TSP-1 in only one cell line, and no effect on VEGF-A or PEDF. AA showed a similar lack of effect.

**Conclusions:**

Since TA and AA do not stimulate uveal melanoma cell growth, it seems to be safe to use these drugs to treat radiation retinopathy after irradiation for uveal melanoma. Additional experiments using more cell lines or primary tumor cell cultures are needed to validate this conclusion. Furthermore, the results of our study suggest that TA does not exert its antileakage effect through downregulation of VEGF-A or upregulation of TSP-1 or PEDF in uveal melanoma cell lines. It is possible that TA and AA influence these pro- and antiangiogenic factors only under hypoxic circumstances. Further investigation is needed.

## Introduction

Uveal melanoma (UM) is the most common primary intraocular tumor in adults, with an incidence of approximately 5–7 per 1,000,000 in Caucasian populations [1-4]. The five-year tumor-related survival rate is about 70%–80% [5,6], and eventually about 50% of patients will die from the disease [7,8]. Because the eye lacks lymphatic vessels, the route of dissemination is almost exclusively hematogenous. The most common site of metastasis formation is the liver [9,10]. Predictors of survival for UM patients are identified by histologic cell type, tumor diameter, tumor location, age, gender [11], and cytogenetic parameters. Loss of chromosome 3 is one of the most significant predictors for UM-related deaths [12,13]. There are different modes of therapy available for treating a primary UM. Enucleation remains a common treatment for large tumors. Small and medium-sized tumors with a prominence <8 mm can be treated with proton beam therapy, local resection, or radiation [14,15]. Radiotherapy is often combined with transpupillary thermotherapy, which may also allow treatment of larger tumors [16,17]. Radiotherapy, however, not only damages UM cells, but also normal healthy cells located in the immediate area surrounding the tumor. Capillary endothelial cells are especially sensitive to radiation and presumably die directly or later by mitotic death [18]. Capillary endothelial cell loss causes changes in the structure and permeability of the vessels in the affected area. This leads to an occlusive microangiopathy, clinically known as radiation retinopathy. A variety of sight-threatening manifestations are associated with radiation retinopathy, such as macular edema and neovascularization [19]. The onset of radiation retinopathy occurs on average 26 months after radioactive plaque therapy [20]. The cause of this delay is not quite clear, although duration of the endothelial cell cycle and delayed mitotic cell death might be an explanation [18]. Gunduz et al. [21] found that approximately 5% of patients treated by radioactive plaque developed radiation retinopathy after one year. After five years this percentage rose to about 40%.

At the moment, there is no established effective treatment for radiation retinopathy and its complications, although laser photocoagulation has shown some beneficial effect [20]. Finger and Chin [22] demonstrated that antiangiogenic agents, such as Avastin, have a beneficial effect.

Triamcinolone acetonide (TA) is a glucocorticoid that has already been shown to improve vision for a few months in patients with macular edema associated with diabetic retinopathy [23-25] and in patients with exudative age-related macular degeneration (ARMD) [26]. Although the causal mechanisms underlying these diseases differ, diabetic retinopathy, exudative ARMD, and radiation retinopathy share the same sight-threatening complications, such as macular edema and neovascularization. A recent study reported a temporary positive effect of intravitreal TA injections in a group of 31 patients with radiation maculopathy [27].

While TA may be a suitable drug to treat complications of radiation retinopathy, its effect on UM cells is unknown. The possibility that there are living UM cells in the choroid of eyes treated with radiotherapy cannot be excluded [28]; local recurrences do develop [29]. We therefore set out to study the effect of TA on the proliferation of UM cells before using this drug in patients with radiation retinopathy. To our knowledge, this has not been investigated previously.

TA has been shown to have an antiangiogenic effect [30-32], though the mechanism by which this effect comes about is not clear. The extent of tumor angiogenesis is determined by the balance between proangiogenic and antiangiogenic molecules, released by both tumor cells and surrounding cells [33]. The formation of new vessels is a process involving endothelial cells; tumor cells that lack a sufficient blood supply are most likely the incentive of angiogenesis. Vascular endothelial growth factor-A (VEGF-A) plays an important role in angiogenesis, regulating vasopermeability and the proliferation and migration of endothelial cells [34]. VEGF-A seems also to be the key mediator in ocular vessel diseases as well as in tumor angiogenesis [35].

We also studied two inhibitors of angiogenesis, pigment epithelium-derived factor (PEDF) and thrombospondin-1 (TSP-1). Various studies have documented that PEDF inhibits angiogenesis in the eye [36,37], whereas TSP-1 has been shown to inhibit cutaneous melanoma progression by suppressing tumor vessel formation [38].

One of the disadvantages of TA, however, is that it causes ocular hypertension in about 30% of the cases [39] and may lead to glaucomatous damage. A new steroid-derived substance, anecortave acetate (AA), an angiostatic cortisone, has been developed to be devoid of corticosteroid side effects such as ocular hypertension [40]. Hence, AA may be a good alternative to TA. A first study of AA administered in a juxtascleral depot to treat subfoveal choroidal neovascularization in ARMD showed good results in the prevention of further vessel development [41]. We therefore analyzed the effect of both TA as well as AA on proliferation, expression and production of pro- and antiangiogenic factors.

## Methods

### Cell lines

Three well described cell lines were selected on the basis of known differences in VEGF-A production (low, medium, high) to perform our experiments: Mel 285, 92–1 and OCM-1. Cell line OCM-1 was kindly provided by Dr. J. Kan-Mitchell (Karmanos Cancer Institute, Detroit, MI) [43]. Cell line 92–1 was established in our laboratory [42]. Mel 285 was a generous gift of Dr. B.R. Ksander (Schepens Eye Research Institute, Boston, MA) [44]. Cell lines Mel 285 and 92–1 were cultured in Roswell Park Memorial Institute (RPMI) 1640 (Gibco Life Technologies, Breda, the Netherlands) to which 10% fetal calf serum, 2% glutamine (Gibco), and 2% penicillin and streptomycin (Gibco) were added. OCM-1 was cultured in Dulbecco's Modified Eagle's Medium (DMEM; Gibco) with 10% fetal calf serum and 2% penicillin and streptomycin (Gibco). Cultures were passaged every three to four days.

### Triamcinolone acetonide and AL-4940 preparations

We used triamcinolone acetonide T6501 (Sigma-Aldrich, St. Louis, MO). The recommended intravitreal dose of TA is 4 mg (0.1 ml) [27]. The dose to which the UM cells are expected to be exposed in vivo after injection of TA is 1 mg/ml. Unfortunately, due to massive TA crystal deposits the maximal tolerated dose in vitro was 0.04 mg/ml. Ten mg TA was suspended in 230 μl methanol, producing a 100 mM stock suspension. This stock was serially diluted with medium to concentrations of 10 μM and 100 μM. Extra methanol was added to the 10 μM suspension to obtain a concentration of 0.1% methanol, similar to the 100 μM suspension. As a control, we used solutions of 0.1% methanol in medium. We used 9(11)-dehydrocortisol (AL-4940–06; Alcon Laboratories, Fort Worth, TX) instead of AA. In vivo AA is rapidly deacetylated into AL-4940, and AL-4940 is therefore the predominantly present active form [45]. A 10 mM stock solution was prepared by dissolving 5.4 mg AL-4940 in 1.6 ml dimethyl sulfoxide (DMSO). This stock was serially diluted with medium to concentrations of 0.1, 1.0, and 10 μM. A solution of 0.1% DMSO was used as control.

### Triamcinolone acetonide administration

Growth assays were performed in triplicate. For each assay, 64 wells per cell line were filled with 1x10^4^ UM cells per well in 1 ml medium, using 24 well plates. Cells were allowed to settle for 24 h. After 24 h, the regular medium was replaced by 1 ml of one of the following media: RPMI/DMEM (control), RPMI/DMEM with methanol (second control), 10 μM TA suspension, or 100 μM TA suspension; for the AL-4940 assay: RPMI (control), RPMI with DMSO (second control), 0.1 μM AL-4940 solution, 1.0 μM AL-4940 solution, or 10 μM AL-4940 solution. Cells were cultured in an incubator at 37 °C and 5.0% CO_2_.

### Cell proliferation and cytotoxicity

Four, six, eight, and ten days after medium replacement, cells were counted or replaced with fresh specific media. Cells from four wells of each cell line at one time point were pooled to collect a high enough number of cells to obtain a reliable count. Cell death was determined by trypan blue dye-exclusion using a Bürker counting chamber (Omnilabo, Breda, the Netherlands). In addition, cell proliferation was measured by mitochondrial function using the WST-1 assay (Roche Diagnostics, Indianapolis, IN), as previously described [46]. In short, 96 well plates were allotted 1250 UM cells per well and either filled with regular medium (control) or 100 μM TA suspension. Absorbance was measured at 450 nm (n=8) on a multiwell spectrophotometer (Perkin Elmer, Wellesley, MA).

### VEGF-A and PEDF production

In the supernatant obtained on day eight, VEGF-A and PEDF concentrations were determined using commercial solid phase sandwich enzyme-linked immunosorbent assay kits. For VEGF-A, we used a human VEGF-A ELISA immunoassay kit (Biosource, Camarillo, CA), and for PEDF, we used a PEDF Sandwich ELISA antigen detection kit (BioProducts MD, Middletown, MD).

### Real-time Quantitative RT–PCR analysis

Several different gene mRNA expressions were analyzed by real-time quantitative reverse transcriptase–polymerase chain reaction (RT–PCR). RNA was isolated on day 8 in one TA assay, using an Rneasy® Mini Kit (Qiagen, Valencia, CA). RNA samples were stored at −80 °C until further processing, when approximately 1 μg of RNA per sample was reverse-transcribed using the iScript cDNA synthesis kit (Bio-Rad, Hercules, CA). The solutions (20 μl) were diluted by adding sterile water until the volume reached a total of 100 μl. In 96 wells, 2 μl of this solution was added to a 15 μl solution of iQ SYBR Green Supermix, forward and reverse primers (10 μM solutions) for VEGF-A, PEDF, TSP-1, beta-actin (β-ACTIN), hypoxantine phosphoribosyltransferase (HRPT), or ribosomal protein S11 (RPS-11), and sterile water (volume ratio respectively 10:1:1:8). The primers for all genes under study were designed with the Primer Express software (PE Applied Biosystems; Foster city, CA; Table 1) [47]. A quantitative analysis of the samples was then performed for all genes by real-time quantitative RT–PCR in a MyiQ iCycler real-time PCR system (Bio-Rad). An accepted method to correct the sample-to-sample variation when determining gene expression is to select a cellular housekeeping gene that serves as an endogenous control, against which the target gene expression levels can be normalized [48]. RPS-11 is a housekeeping gene that has been recently used to normalize gene expression in UM cells [47]. β-actin is a relatively stable cytoskeletal protein generally thought to be present at a constant level in cells, regardless of experimental treatment or technical procedure [49]. HPRT, an enzyme in purine metabolism, is reported as a constitutively expressed housekeeping gene [49,50].

**Table 1:** Primer sequences of the genes studied in the RT–PCR assay

**Primer**	**Gene**	**Forward primer (5’-3’)**	**Reverse primer (5’-3’)**
Endogenous controls	*RPS-11*	AAGCAGCCGACCATCTTTCA	CGGGAGCTTCTCCTTGCC
	*HRPT*	CGAGATGTGATGAAGGAGATGG	GCAGGTCAGCAAAGAATTTATAGC
	*β-ACTIN*	CGGGACCTGACTGACTACCTC	CTCCTTAATGTCACGCACGATTT
Genes under study	*VEGF-A*	GCCCTTGCCTTGCTGCTCTACC	GTGATGATTCTGCCTCCTCCTTC
	*PEDF*	AGCATTCTCCTTCTCGGTGTGG	CCTCACGGTCCTCTCTTCATCC
	*TSP-1*	AGGTCTTCAGCGTGGTGTCC	ACAAACAGGGTGATGCTCTTCC

Primer sequences of the control and studied genes that were used in real-time quantitative RT-PCR assay to determine level of expression in UM cell lines. Abbreviations: β-ACTIN is beta-actin; HPRT is hypoxantine phosphoribosyltransferase; RPS-11 is ribosomal protein S11.

The PCR reaction settings were 95 °C for 3 min, then 40 cycles at 95 °C for 30 s and 60 °C for 30 s, then 95 °C for 1 min and 60 °C for 1 min. Using a control, we calculated assigned relative quantity for any sample for all genes as follows:

Relative Quantity_sample (Gene X)_=E_Gene X_^[C^_T_^ (Control) – C^_T_^ (sample)]^

where E represents efficiency of primer or (probe) set, C_T_ ^(Control)^ equals the average C_T_ for the sample which has been assigned as a control, and C_T_ ^(sample)^ is the average C_T_ for the sample. This is referred to as normalized expression [51].

### Statistical analysis

Data are expressed as the mean±standard deviation. Student’s *t*-test was used to determine whether there were statistically significant differences between treatment groups in the cell viability assay. A p<0.05 was considered statistically significant.

## Results

### Influence of TA and AA on UM cell proliferation

TA and AA may be potential drugs to treat radiation retinopathy. However harmful side effects such as proliferation of UM cells have to be ruled out.

**Figure 1:**
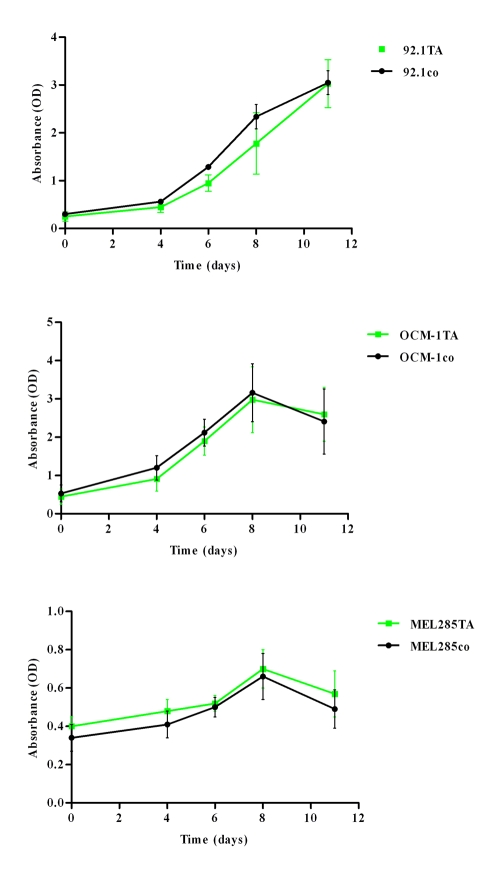
Effect of TA on uveal melanoma cell proliferation. Cells were treated with four different suspensions: Park Memorial Institute/Dulbecco’s Modified Eagle Medium (RPMI/DMEM; control), RPMI/DMEM with methanol (second control), 10 mM TA suspension, or 100 mM TA suspension. Cell proliferation was determined by a WST-1 assay (absorbance (OD) and the mean±SD is provided for each time point (n=8)). No substantial difference was seen in proliferation of uveal melanoma cell lines between TA treatment and control (cell line 92.1 (p=0.755), OCM-1 (p=0.844), and Mel285 (p=0.487)). Abbreviations: OD is optical density; TA is triamcinolone acetonide; CO is control.

Treatment of UM cells with TA did not induce cell death as measured by the Bürker counting chamber (data not shown). The WST-1 assay demonstrated no significant effect of TA treatment on cell proliferation of the UM cell lines 92.1 (p=0.755), OCM-1 (p=0.844), and Mel285 (p=0.487; Figure 1). AA showed similar results. TA and AA had no toxic effect on UM cells, as there were no differences in the percentages of dead cells between the different treatments (data not shown).

### VEGF-A and PEDF production

To investigate any direct pro- or antiangiogenic effects of TA and AA on UM cells, we studied the influence of these drugs on VEGF-A and PEDF production of three UM cell lines using an ELISA on culture supernatant. Since cell numbers per well differed considerably between some treatments and experiments, we corrected for the number of cells by dividing the concentration of VEGF-A or PEDF measured in the supernatant by the amount of cells present in the same well. All cell lines produced VEGF-A and PEDF, but clear differences were observed in the amounts: OCM-1 produced the highest levels of VEGF-A (50 pg/ml/10^4^ cells); 92–1 produced approximately one-fifth of this amount (10 pg/ml/10^4^ cells); and Mel 285 produced the lowest amount of VEGF-A (5 pg/ml/10^4^ cells).

**Figure 2:**
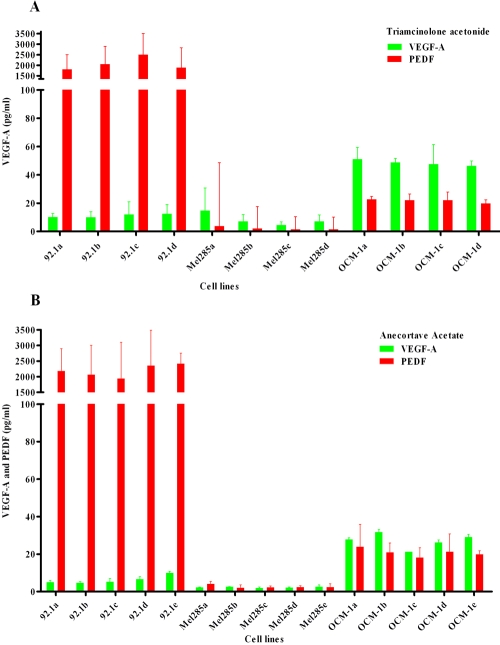
Production of VEGF-A and PEDF in three different UM cell lines exposed to various TA and AA doses. Cells were treated with four or five different suspensions. **A:** For triamcinolone acetonide (TA) experiments, we used either Park Memorial Institute/Dulbecco’s Modified Eagle Medium (RPMI/DMEM; control), RPMI/DMEM with methanol (second control), a 10 mM TA suspension, or a 100 mM TA suspension ; **B:** For anecortave acetate (AA) experiments, we used either RPMI (control), RPMI with dimethyl sulfoxide (DMSO; second control), 0.1 mM AL-4940 solution, 1.0 mM AL-4940 solution, or 10 mM AL-4940 solution. The level of vascular endothelial growth factor-A (VEGF-A) protein (n=2) is shown in pg/ml (mean±SD). All cell lines produced VEGF-A and pigment epithelium-derived factor (PEDF), but clear differences were observed: OCM-1 produced the highest levels of VEGF-A, while cell line Mel 285 produced the lowest amount of VEGF-A. Cell line 92–1 produced large amounts of PEDF (>2000 pg/ml). Mel 285 produced scant PEDF. Addition of TA or AA to the cell cultures had no effect on either VEGF-A or PEDF production.

Cell line 92–1 produced large amounts of PEDF (2.000 pg/ml/10^4^ cells), around 100 times the amount produced by cell line OCM-1 (20 pg/ml/10^4^ cells). Mel 285 produced very little PEDF (3 pg/ml/10^4^ cells). Addition of TA or AA to the cell cultures had no effect on either VEGF-A or PEDF production (Figure 2A,B).

### VEGF-A, PEDF, and TSP-1 expression

**Figure 3:**
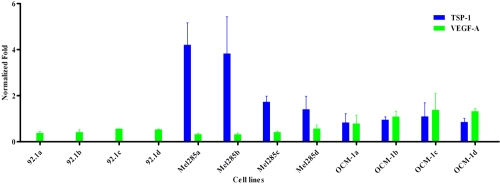
The effect of TA (two doses) on gene expression of VEGF-A and TSP-1 in three different uveal melanoma cell lines. Cells were treated with four different suspensions: Park Memorial Institute/Dulbecco’s Modified Eagle Medium (RPMI/DMEM; control), RPMI/DMEM with methanol (second control), 10 mM triamcinolone acetonide (TA) suspension, or 100 mM TA suspension. Expression levels of vascular endothelial growth factor-A (VEGF-A) and thrombospondin-1 (TSP-1) are normalized against two endogenous controls (normalized fold; mean±SD). Experiments were performed twice. VEGF-A was expressed in all cell lines at variable levels. TA treatment had no major (>1.0 fold) effect on VEGF-A expression. TSP-1 was not expressed in 92.1. Mel 285 showed a decreased TSP-1 expression (time-fold difference=1.5–2.9) after treatment with TA.

We determined by real-time quantitative RT–PCR whether TA or AA had any effect on several genes in UM cell lines that were associated with angiogenesis. VEGF-A was expressed in all cell lines at variable levels. TA treatment had no major (>1.0 time fold difference) effect on VEGF-A expression. On the contrary, TA gave a dose-dependent reduction of TSP-1 expression in Mel285 (time fold difference=1.5–2.9). TSP-1 was hardly expressed in 92.1 (Figure 3). AA had no significant effect either on VEGF-A or TSP-1 expression. PEDF is highly expressed in cell line 92–1 and only to a minimal extend in the other cell lines. PEDF expression in cell line 92–1 was not affected by TA or AA (data not shown).

## Discussion

Our results showed no apparent stimulating or inhibiting effect of TA on UM cell proliferation in the concentrations used in this study [27]. The effect of TA on UM cell lines has not yet been investigated, but the effect of TA on other cell types has been the object of some studies. For instance, TA was found to inhibit the growth of retinal pigment epithelium (RPE) when ARPE 19 cells were treated with concentrations ranging from 1 μM to 2.3 mM, but stimulated growth at a concentration of 1 nM [52,53]. Another study, using bovine retinal endothelial cells, found that TA at a concentration of 115 μM had no effect on growth; concentrations ranging from 230 μM to 4.6 mM had an inhibiting effect, and concentrations of 6.8 mM and higher were cytotoxic [54]. Likewise, in vitro incubation of RPE with corticosteroids induced a specific and dose-dependent reduction of cell viability. These toxic events were not associated with the anti-inflammatory activity of these compounds but depended on the hydrosolubility of their formulation [55].

While previous studies on RPE cells demonstrated an inhibition of VEGF production and expression by TA, we found no effect of TA on VEGF-A production or expression in UM cells. For instance, TA reduced VEGF expression of ARPE19 cells under oxidative stress [53] and inhibited cobalt-stimulated VEGF production in Müller cells, probably by a destabilization of VEGF mRNA [56]. The lack of inhibition of TA on VEGF-A production and expression in our study could perhaps be explained by the absence of specific stimuli that would upregulate VEGF-A production and expression, such as oxidative stress or cobalt administration. In the study by Sears et al. [56], only cobalt-stimulated VEGF production was inhibited by TA, while basal VEGF production was not affected. Similarly, another study showed no alteration in VEGF expression by TA in a rat retina when no special stress was applied [57].We are currently performing experiments to determine the effect of TA and other drugs on UM cells under stressful conditions, such as hypoxia.

PEDF inhibits angiogenesis by inducing apoptosis in endothelial cells that try to form new vessels [58,59]. Besides the separate levels of VEGF-A and PEDF, the balance between the two seems to be decisive in whether or not angiogenesis takes place [60]. Yang et al. previously demonstrated expression of PEDF in UM cell lines. Furthermore, they observed an inhibitory effect of angiostatin on the ratio of VEGF:PEDF mRNA levels in vitro [59]. Likewise, Mel 285, 92–1, and OCM-1 all produced PEDF in our experiments. We observed no effect of TA on the production of PEDF by any of the UM cell lines. Therefore, stimulation of production of PEDF by UM cells seems not to be the antiangiogenic working mechanism of this drug under normal circumstances. The high expression of PEDF by cell line 92–1 was unexpected, since PEDF has been shown to work as a tumor growth inhibitor through antiangiogenic mechanisms [61-63] and cell line 92–1 was derived from a highly malignant tumor [42]. However, there are reports that melanocytic tumors have a relatively high expression of PEDF [64]. Also, PEDF might have a biphasic activity, inhibiting angiogenesis at normal amounts, but stimulating angiogenesis at high concentrations [65].

The balance between VEGF-A and PEDF, as we have described, seems also to be influenced by TSP-1 in that it inhibits endothelial cell migration and proliferation and also induces apoptosis [66]. In cell line Mel 285, TSP-1 expression was about twofold inhibited in cells grown in TA compared to the control cells. Previous reports on the effect of TA on the expression of TSP-1 could not be found, though some effects of other corticosteroids have been reported. Hydrocortisone upregulated TSP-1 expression in a glioma cell line [67], and dexamethasone increased TSP-1 expression in a murine trofoblast-like cell culture [68]. The results of Mel 285 do not agree with this, and may indicate an unexpected effect of TA. TSP-1 expression of cell line 92–1 was very low, possibly due to inactivation of this gene in the same manner as described for hypermethylated p16 (INK4a) [69].

Parallel experiments with AA, designed to eliminate any possible side effects of the glucocorticoid fraction, showed similar results as with TA. There seems to be no differential effect of this angiostatic cortisone compared to TA on potentially still viable UM cells in the choroid after radiotherapy; both drugs have hardly any effect at all.

The risk of an increase in local recurrences of UM by TA or AA in the treatment of radiation retinopathy by direct stimulation of UM cells seems to be low. We based these conclusions on a restricted number of cell lines, and extrapolation to the in vivo situation is by definition limited. In addition, the clinical dose of TA could not be investigated due to massive crystal deposits. Perhaps a certain threshold has to be reached before TA can demonstrate any effect. Nonetheless, our results suggest that TA does not exert any antiangiogenic effect through influencing basal VEGF-A, PEDF, TSP-1 production or expression in UM cells. However, influence of hypoxia and stimulation of these cell lines by VEGF or FGF2 or by antiangiogenic factors is possible and is under investigation.

Furthermore, there are many other pro- and antiangiogenic factors that could be altered by TA. Plasminogen activator inhibitor type-1 for instance, has been implicated in metastasis formation and tumor-associated angiogenesis in UM [70], and would be an interesting candidate for further studies. These factors will need further investigation.
